# Optimization of dose distributions of target volumes and organs at risk during stereotactic body radiation therapy for pancreatic cancer with dose-limiting auto-shells

**DOI:** 10.1186/s13014-018-0956-7

**Published:** 2018-01-22

**Authors:** Yangsen Cao, Xiaofei Zhu, Xiaoping Ju, Yongming Liu, Chunshan Yu, Yongjian Sun, Zhitao Dai, Xueling Guo, Huojun Zhang

**Affiliations:** Department of Radiation Oncology, Changhai Hospital affiliated to Navy Medical University, 168 Changhai Road, Shanghai, 200433 China

**Keywords:** Auto-shells, Stereotactic body radiation therapy, Pancreatic cancer, Dose limiting

## Abstract

**Background:**

To identify optimization of dose distributions of target volumes and decrease of radiation doses to normal tissues during stereotactic body radiation therapy (SBRT) for pancreatic cancer with dose-limiting auto-shells.

**Methods:**

With the same prescription dose, dose constraints of normal organs and calculation algorithm, treatment plans of each eligible patient were re-generated with 3 shells, 5 shells and 7 shells, respectively. The prescription isodose line and beam number of each patient in 3-shell, 5-shell and 7-shell plan remained the same. Hence, a triplet data set of dosimetric parameters was generated and analyzed.

**Results:**

As the increase of shell number, the conformal index, volumes encompassed by 100% prescription isodose line and 30% prescription isodose line significantly decreased. The new conformal index was higher in 3-shell group than that in 5-shell and 7-shell group. A sharper dose fall-off was found in 5-shell and 7-shell group compared to 3-shell group. And the tumor coverage in 7-shell was better than that of 3-shell and 5-shell. Lower D_5cc_ of the intestine, D_10cc_ of the stomach, D_max_ of the spinal cord and smaller V_10_ of the spleen was confirmed in 7-shell group compared to 3-shell group.

**Conclusions:**

More conformal dose distributions of target volumes and lower radiation doses to normal organs could be performed with the increase of dose-limiting auto-shells, which may be more beneficial to potential critical organs without established dose constraints.

## Background

High doses may be required to achieve local control attributable to inherent radio-resistance of pancreatic cancer. However, the abutting organs at risk (OAR), including the stomach, duodenum and bowel, limit the prescription dose because of radio-sensitivity. Due to the advances in the radiotherapy, the nonisocentric and noncoplanar technique of stereotactic body radiation therapy (SBRT) could deliver precise radiation with high local dose conformation, precise target localization [1] with motion compensation strategies [2, 3] and facilitate quick dose fall-off outside the tumor volume [4, 5].

Nevertheless, treatment planning is strongly user-dependent since optimization parameters were preselected [6, 7]. Hence, a well-balanced plan should be based on the ALARA (as-low-as-reasonably-achievable) principle, and is generally preferable with extreme quality in only a few categories as long as any strict critical structure limits are not violated. Owing to the normal tissues in close proximity to the pancreas, a plan with trade-offs, if clinical justified, among all planning objectives is required.

Traditionally, collimators are nontrivial for controlling dose conformity and dose fall-off. While multiple fixed collimators may allow better conformity and fewer hot spots around the tumor boundary than a single one, but it is time-consuming and a large number of collimators could even impair plan quality. The Iris collimator can reduce treatment time [8], but may not necessarily lead to better dosimetric plan quality [9]. Also, a larger tumor volume indicated a larger aperture collimator, contributing to a larger penumbra and slow dose fall-off, detrimental to normal organs.

Apart from the collimator, shell structures are also used for optimizing dose conformity and reducing skin entry doses as well as hot spots outside the target area. Furthermore, in addition to constraints of OARs, auto-shell is required to control dose gradients around the target volume, but without too much compromise of target volume coverage. Therefore, the selection of the suited number of shell structures and distances between each shell is pivotal.

As a result, the aim of our study was to implement dose-limiting auto-shells in the SBRT for pancreatic cancer to minimize doses of OAR and non-critical organs and further optimize dose distributions of target volumes with Sequential Multi-Objective Optimization.

## Methods

### Eligibility

Medical records of patients with pancreatic cancer receiving SBRT in our center from October 2016 to April 2017 were reviewed. All patients received clinical examinations and abdominal CT or MRI scanning. The inclusion criteria were as follows: diagnose confirmed by pathological examinations; locally advanced pancreatic cancer; patients with resectable or borderline resectable cancer intolerant of surgical resections; age ranging from 18 to 75 years old; tumor located in the pancreatic head with maximum diameter < 5 cm; ECOG ≤2; all patients receiving the same prescription dose. The following exclusion criteria were used: patients with a history of radiotherapy prior to the SBRT; metastatic pancreatic cancer.

### Robotic radiosurgery system and treatment planning system

SBRT was delivered via CyberKnife, an image-guided frameless stereotactic robotic radiosurgery system (Accuray Corporation, Sunnyvale CA). The treatment planning process was carried out with a dedicated treatment planning system, Multiplan version 4.0.2 (Accuray Inc.).

The sequential method was applied for all plans in the investigation. The inverse treatment-planning algorithm was performed to maximize the minimum dose to target volume or the mean dose, known as “optimize coverage (OCO)” in the system. The upper bounding constraints of the OAR were restricted to the following doses during the optimization (volume of interest limits, VOI limits): spinal cord: 3Gy; stomach: 15Gy; intestine: 14Gy; duodenum: 14Gy. In order to minimize the maximum doses to the critical structures, the above limits were stricter than those reported in the American Association of Physicists in Medicine guidelines in TG-101 [10]. The optimization of monitor unit (MU) was performed as follows: total MU: 90,000; max MU per beam: 500; max MU per node: 1500. This could reduce isodose lines showing up as streaks in the direction of beam entry points, and hot spots in the vicinity of the beam entry points just below the skin surface [11].

### Target delineations

The procedure of CyberKnife was similar to our previous study [12, 13]. Gross tumor volume (GTV) was delineated as a radiographically evident gross disease by contrast CT acquired from the portal-venous phase. At the discretion of the physician, clinical target volume (CTV) encompassing areas of the potential subclinical disease spread was also designated. In most cases, the CTV equaled GTV. A 2–5 mm expansion margin was included to determine the planning target volume (PTV). When the tumor was adjacent to critical organs, the expansion of PTV outside of CTV in this direction should be avoided. Therefore, the margin expansion was allowed to be nonuniform. References of normal tissue constraints were according to the American Association of Physicists in Medicine guidelines in TG-101 [10].

### Auto-shells planning

Each patient treatment plan was re-generated with 3 shells, 5 shells and 7 shells, respectively. All the patients received the same prescription dose (37.5Gy/5f). At least 90% of PTV should be covered by the prescription dose. The prescription isodose line was limited to 70–80%, which would restrict the tumor D_max_. The prescription isodose line of each patient in 3-shell, 5-shell and 7-shell plan was the same. Shells were determined based on the expansions of PTV margins (Details shown in Table 1). The dose limitations between every two shells were assigned (Table 2) in order to obtain desired dose fall-off without compromising coverage significantly. The distance from the PTV margin to each shell was 5 mm, 15 mm and 30 mm in 3-shell group, respectively; 2 mm, 5 mm, 15 mm, 30 mm and 60 mm in 5-shell group, respectively; 2 mm, 5 mm, 10 mm, 15 mm, 30 mm, 60 mm and 100 mm in 7-shell group, respectively. Beam reduction in the system was utilized to restrict beam numbers, remove low-weighted beams and reoptimize with the remaining beams to keep the plan quality, which resulted in ensuring the same number of beams in the three plans. The plans were calculated by Monte Carlo algorithm with high resolution and the uncertainty was 1%.

**Table 1:** Generation of shells based on the expansions of PTV margins

	Distance from each shell to the PTV margin (mm)						
	1st shell	2nd shell	3rd shell	4th shell	5th shell	6th shell	7th shell
3-shell group	5	15	30	–	–	–	–
5-shell group	2	5	15	30	60	–	–
7-shell group	2	5	10	15	30	60	100

**Table 2:** Dose limitations between every two shells

	Dose limitations (Gy)						
	PTV margin to 1st shell	1st shell to 2nd shell	2nd shell to 3th shell	3th shell to 4th shell	4th shell to 5th shell	5th shell to 6th shell	6th shell to 7th shell
3-shell group	26	17	9	–	–	–	–
5-shell group	32	26	17	9	6	–	–
7-shell group	32	26	21	17	9	6	5

### Statistical analysis

Parameters selected for evaluation of doses of target volumes included conformity index (CI), new conformity index (nCI), heterogeneity index (HI), gradient index (GI), coverage, volumes encompassed by 100% isodose line (100% PD-V), volumes encompassed by 50% isodose line (50% PD-V) and volumes encompassed by 30% isodose line (30% PD-V). Crucially, the formula of calculation of CI was: $\mathrm{CI}=\frac{prescription\ isodose\ volume\ \left( PIV,{cm}^3\right)}{tumor\ volume\ encompassed\ prescription\ isodose\ line\ \left( TIV,{cm}^3\right)}$. The nCI was calculated as follows: $\mathrm{nCI}=\frac{CI}{coverage}$, in which coverage was defined as the ratio of target volumes covered with prescription dose to the target volume. The HI was determined by the following formula: $\mathrm{HI}=\frac{D_{max}}{R_{xDose\kern0.5em }}$, in which R_xDose_ was the prescription dose. The definition of GI was the ratio of the volume of half the prescription isodose to the volume of the prescription isodose [14]. Evaluations of doses to OAR included the maximum dose (D_max_) and volume doses. D_max_ was defined as the dose of a 0.035-cc or less. The dose volume of the intestine, stomach and spinal cord was the dose of a 5-cc volume (D_5cc_), 10-cc volume (D_10cc_) and 0.35-cc volume (D_0.35cc_). Doses of a 5-cc and a 10-cc volume were investigated in the case of the duodenum. In addition to these OAR, doses to the spleen were also studied, including the mean dose (D_mean_), the dose of a half spleen volume (D_50%_) and the volume receiving 5Gy (V_5_) and 10Gy (V_10_). However, 2 patients had splenectomy. Hence, dose evaluations were only performed in 18 patients.

Comparisons between parameters in different shell groups were based on analysis of variance (ANOVA) for a randomized block design. Fisher’s least significance difference test (LSD) was performed when significant differences were found in the three groups after analysis of variance. Results were deemed significant at *P* <  0.05. Statistical analyses were performed using IBM SPSS version 22.0 (SPSS Inc., Armonk, NY).

## Results

### Patient characteristics

A total of 20 patients were enrolled in the investigation. The median age was 64.5 years old. The prescription dose was 37.5Gy/5f (BED_10_ = 65.625Gy, biological effective dose, α/β = 10). The median GTV and PTV was 26.95cm^3^ and 36.33cm^3^, respectively. The beam number ranged from 150 to 210 and the median was 186.

### Evaluations of dose distributions of PTV

With the increase of shell number, the CI, 100% PD-V and 30% PD-V significantly decreased (CI: 3-shell vs 5-shell: 1.12 ± 0.05 vs 1.10 ± 0.04, *P* <  0.001; 5-shell vs 7-shell: 1.10 ± 0.04 vs 1.09 ± 0.03, *P* = 0.024; 3-shell vs 7-shell: 1.12 ± 0.05 vs 1.09 ± 0.03, *P* <  0.001) (100% PD-V: 3-shell vs 5-shell: 38.92 ± 12.31 vs 38.45 ± 12.85, *P* = 0.036; 5-shell vs 7-shell: 38.45 ± 12.85 vs 37.73 ± 12.62, *P* = 0.002; 3-shell vs 7-shell: 38.92 ± 12.31 vs 37.73 ± 12.62, *P* <  0.001) (30% PD-V: 3-shell vs 5-shell: 344.53 ± 119.93 vs 312.90 ± 110.18, *P* <  0.001; 5-shell vs 7-shell: 312.90 ± 110.18 vs 299.15 ± 106.41, *P* = 0.006; 3-shell vs 7-shell: 344.53 ± 119.93 vs 299.15 ± 106.41, *P* <  0.001). Additionally, there was significant difference between 3-shell and 5-shell, 3-shell and 7-shell with respect to nCI and GI. Furthermore, the 50% PD-V was smaller in 7-shell compared with that of 3-shell and 5-shell. And the tumor coverage in 7-shell was better than that of 3-shell and 5-shell (Table 3).

**Table 3:** Evaluations of dose distributions of PTV

	Group			*P*-value (ANOVA)	*P*-value (LSD, 3-shell vs 5-shell)	*P*-value (LSD, 5-shell vs 7-shell)	*P*-value (LSD, 3-shell vs 7-shell)
Parameter	3-shell	5-shell	7-shell				
CI	1.12 ± 0.05	1.10 ± 0.04	1.09 ± 0.03	< 0.001	< 0.001	0.024	< 0.001
nCI	1.21 ± 0.04	1.19 ± 0.03	1.18 ± 0.03	< 0.001	< 0.001	0.185	< 0.001
HI	1.44 ± 0.01	1.44 ± 0.01	1.44 ± 0.01	1.0	1.0	1.0	1.0
GI	3.03 ± 0.14	3.09 ± 0.13	3.07 ± 0.14	0.012	0.004	0.368	0.037
Coverage (%)	92.63 ± 2.22	92.60 ± 1.83	91.79 ± 1.44	0.016	0.904	0.014	0.010
100%_PD (cc)	38.92 ± 12.31	38.45 ± 12.85	37.73 ± 12.62	< 0.001	0.036	0.002	< 0.001
50%_PD (cc)	117.48 ± 35.83	117.65 ± 36.53	115.09 ± 36.84	< 0.001	0.749	< 0.001	< 0.001
30%_PD (cc)	344.53 ± 119.93	312.90 ± 110.18	299.15 ± 106.41	< 0.001	< 0.001	0.006	< 0.001
MU	54,674.51 ± 13,149.78	54,649.06 ± 13,246.72	54,058.67 ± 12,744.23	0.018	0.914	0.016	0.012

### Evaluations of doses to OAR

The doses to normal tissue were shown in Table 4. After restriction of doses to OAR with different shells, D_5cc_ of the intestine, D_10cc_ of the stomach and D_max_ of the spinal cord was lower in 7-shell than those in 3-shell (D_5cc_ of the intestine: 3-shell vs 7-shell: 15.50 ± 2.10 vs 14.95 ± 2.51, *P* = 0.003; D_10cc_ of the stomach: 3-shell vs 7-shell: 14.32 ± 1.74 vs 13.97 ± 2.23, *P* = 0.020; D_max_ of the spinal cord: 3-shell vs 7-shell: 7.12 ± 1.20 vs 6.63 ± 0.89, *P* = 0.046), though only a trend of decrease of D_10cc_ of the stomach and D_max_ of the spinal cord was found when the number of shell increased.

**Table 4:** Evaluations of dose to OAR

	Group			*P*-value (ANOVA)	*P*-value (LSD, 3-shell vs 5-shell)	*P*-value (LSD, 5-shell vs 7-shell)	*P*-value (LSD, 3-shell vs 7-shell)
Parameter	3-shell	5-shell	7-shell				
Intestine (D_max_)	21.69 ± 3.13	21.28 ± 3.62	21.17 ± 3.64	0.359	0.286	0.769	0.176
Intestine (D_5cc_)	15.50 ± 2.10	15.27 ± 2.51	14.95 ± 2.51	0.011	0.183	0.076	0.003
Stomach (D_max_)	22.14 ± 2.12	22.17 ± 1.66	22.12 ± 2.35	0.976	0.897	0.826	0.928
Stomach (D_10cc_)	14.32 ± 1.74	14.13 ± 2.13	13.97 ± 2.23	0.064	0.183	0.292	0.020
Duodenum (D_max_)	18.23 ± 4.04	18.27 ± 4.11	17.91 ± 4.01	0.274	0.891	0.147	0.187
Duodenum (D_5cc_)	10.54 ± 3.50	10.72 ± 3.55	10.71 ± 3.43	0.63	0.395	0.961	0.422
Duodenum (D_10cc_)	8.76 ± 3.22	8.81 ± 3.30	8.80 ± 3.20	0.968	0.808	0.953	0.854
Spinal cord (D_max_)	7.12 ± 1.20	6.91 ± 1.28	6.63 ± 0.89	0.131	0.374	0.251	0.046
Spinal cord (D_0.35cc_)	6.56 ± 1.05	6.36 ± 1.12	6.17 ± 0.75	0.151	0.321	0.328	0.053
Spleen (D_mean_)	31.99 ± 11.91	30.70 ± 12.03	30.34 ± 11.38	0.989	0.987	0.890	0.903
Spleen (V_5_)	7.47 ± 11.27	5.22 ± 11.88	4.24 ± 11.28	0.851	0.697	0.873	0.584
Spleen (V_10_)	0.005 ± 5.42	0 ± 5.42	0 ± 5.23	0.037	0.339	1.000	0.012
Spleen (D_50%_)	26.60 ± 5.73	26.68 ± 7.66	26.96 ± 5.31	0.903	0.678	0.721	0.954

Crucially, in addition to the conventional OARs, the dose-volume parameters of the spleen were further investigated. There was no significant difference between the three groups regarding D_mean_, V_5_ and D_50%_. Nevertheless, V_10_ was much smaller in 7-shell group than that in 3-shell group.

**Fig. 1:**
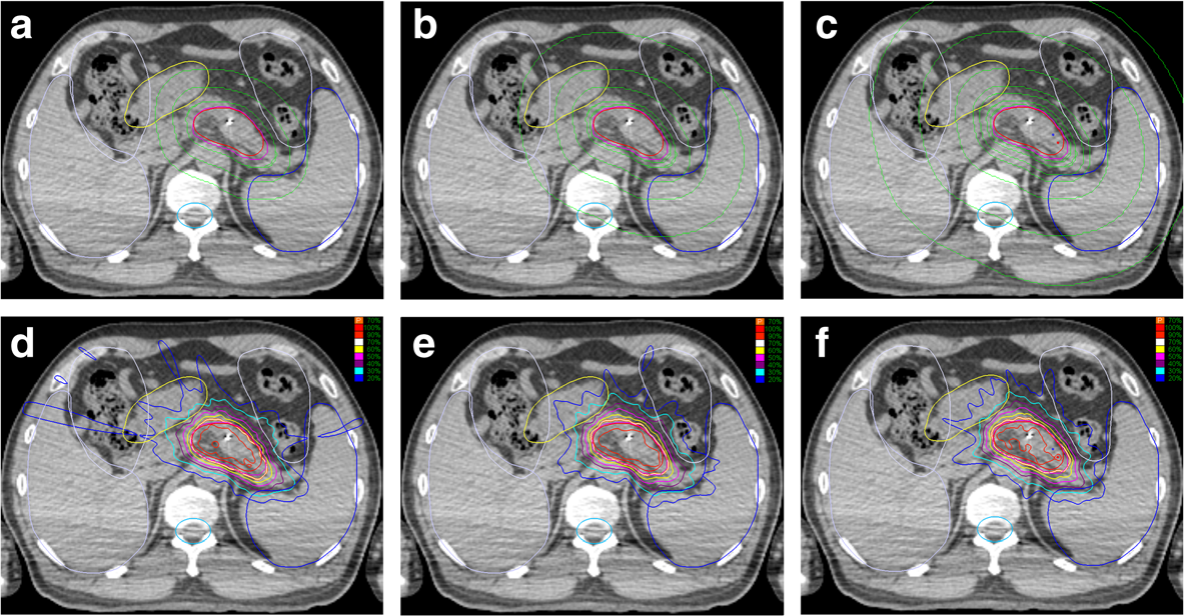
**a** three-shell plan and **d** the dose distribution, **b** five-shell plan and **e** the dose distribution, **c** seven-shell plan and **f** the dose distribution

Given that one patient with a large tumor abutting to the spleen, invading the splenic hilar, the radiation dose was much higher than that for other patients. Therefore, the further analysis of V_10_ precluded the patient was performed. It was also shown that V_10_ was smaller in 7-shell group compared with 3-shell group (3-shell: 0 ± 1.31; 5-shell: 0 ± 1.30; 7-shell: 0 ± 0.82; 3-shell vs 5-shell, *P* = 0.367; 5-shell vs 7-shell: *P* = 0.156; 3-shell vs 7-shell: *P* = 0.024). An exemplary image showing the comparison of dose contributions between the three shells, five shells and seven shells plan was illustrated (Fig. 1).

## Discussion

Complex tumor shapes or lesions adjacent to critical organs were challenging to treatment plans, which however did not indicate multiple calibration of structures or complicated optimization steps [15]. Therefore, even a high-quality treatment plan, short and simple optimization scripts may be appropriate [14]. Besides, manual tuning parameters combined with iterative optimization with different scripts were based on trade-offs between planning objectives, which were determined by clinical preferences and should be prioritized. Hence, a well-balanced plan required emphasis on several planning constraints and aims without any compromise of other vital goals. In our study, all of the treatment plans were generated with pre-selection of constraints, some of which were even stricter than those recommended, and optimization of PTV coverage (OCO), in order to meanwhile comply with ALARA. All other constraints remained unchangeable to explore the influence of shell structures on dose distributions.

The results of our study indicate that with the increase of the shells, CI, volumes encompassed by different isodose lines, doses to OAR with a certain volume and V_10_ of the spleen decreased while dose conformity was better. In the Multiplan^®^ System, auto-shells are generated at the expansion of different diameters based on the PTV. Typically, the generation of auto-shells could be performed with a unique dilation value per direction, or with one value to all directions for symmetric dilations. Auto-shells are used to control dose distribution both close to and far away from a target. An auto-shell close to a target can modify conformity, while a shell distant to a target can be used to control low dose distributions or remote hot spots. Additionally, for those normal tissues not contoured as OAR, which were deemed possibly invulnerable to the radiation, hot spots could be avoided by auto-shells.

In previous studies, it was suggested that a shell structure of 1-3 mm was used to limit the high dose conformity, while two auto-shells with a distance between each other of 5-10 mm for the optimization of the prescription dose conformity and 15-20 mm for the optimization of proximate dose fall-off. Additionally, a shell structure of 30-50 mm was employed to limit the skin doses and hot spots outside the proximate target region [9]. Therefore, the number and distance of shell structures should be adjusted according to the target volume, clinical justifications and planning goals. Furthermore, Schlaefer et al. [15] concluded that an additional shell structure with a distance of 5 mm to the PTV should be used to control dose gradient around the PTV. In this study, the distances between each shell were similar to those reported.

Yoon et al. [16] has reported clinical practice of different shells for brain metastases. In their study, the original CyberKnife plans were produced using one to three dose-limiting auto-shells at prescription dose level and low dose levels of 10 to 30% of PD. The modified CyberKnife plans were generated by five dose-limiting shells at prescription dose level, intermediate dose level of 50% of prescription dose, and low dose levels with an optimal shell-dilation size based on their experience. It was shown that there was no difference in CI and tumor coverage between two plans, while the normal tissue volume receiving 50% of prescription dose was significantly decreased in the modified plans compared with the original plans by 1.28-fold, indicating that a steeper dose fall-off could be achieved with the increase of auto-shells.

The previous results were similar to ours. The GI was higher in 5-shell and 7-shell group compared with 3-shell group. The underlying reason may be that the allowed distance for dose fall-off (37.5Gy to 26Gy) from the PTV margin to the first shell was 5 mm in 3-shell group. While in 5-shell and 7-shell group, the dose fall-off (37.5Gy to 26Gy) was divided into two processes: the dose decreased from 37.5Gy to 32Gy within a short distance of 2 mm (from the PTV margin to the first shell), and from 32Gy to 26Gy within a distance of 3 mm (from the first shell to the second shell).

Additionally, it was elucidated that V_10_ of the spleen was significantly lower in 7-shell group than that in 3-shell group, which may suggest that the radiation dose to the spleen was lower in 7-shell group.

## Conclusions

Better conformity, lower radiation doses and smaller low-dose areas of normal organs could be achieved with the increase of dose-limiting auto-shells, though maybe at the cost of tumor coverage. Crucially, for organs without established dose constraints, milder radiation-induced toxicities may be attributable to auto-shells. Typically, protection of the spleen from excessive unintentional irradiation with auto-shells may reduce the incidence of lymphocytopenia, which may be beneficial to prognosis. Therefore, auto-shells could be an option to customize individual plans to ensure adequate normal organs sparing when clinically indicated.

## Abbreviations

100% PD-V: Volumes encompassed by 100% isodose line
30% PD-V: Volumes encompassed by 30% isodose line
50% PD-V: Volumes encompassed by 50% isodose line
ALARA: As-low-as-reasonably-achievable
ANOVA: Analysis of variance
CI: Conformity index
CTV: Clinical target volume
GI: Gradient index
GTV: Gross tumor volume
HI: Heterogeneity index
LSD: Fisher’s least significance difference test
MU: Monitor unit
nCI: new conformity index
OAR: Organs at risk
OCO: Optimize coverage
PTV: Planning target volume
SBRT: Stereotactic body radiation therapy
VOI: Volumes of interest
